# Attitude towards Euthanasia among Medical Students: A Cross-Sectional Study in Hong Kong

**DOI:** 10.3390/ijerph19137697

**Published:** 2022-06-23

**Authors:** Amy Mei-Yin Lau, Eliza Lai-Yi Wong

**Affiliations:** 1 JC School of Public Health and Primary Care, The Chinese University of Hong Kong, Hong Kong SAR, China; laumeiyin23@gmail.com; 2 Centre for Health Systems and Policy Research, JC School of Public Health and Primary Care, The Chinese University of Hong Kong, Hong Kong SAR, China

**Keywords:** euthanasia, medical students, attitudes

## Abstract

Background: With an increasing aging population and heavy medical burden, euthanasia has become a controversial topic in Hong Kong (HK) in recent years. Medical students are future medical professionals who may face novel and evolving ethical dilemmas. Hence, their views on euthanasia are crucial. Objective: To examine the attitudes of medical students towards euthanasia in HK and identify the factors associated with their attitude towards euthanasia. Methods: A questionnaire-based cross-sectional study among medical students in HK was conducted. The online anonymous questionnaires were distributed to all six years of students studying medicine at the Chinese University of Hong Kong (CUHK) and the University of Hong Kong (HKU), who provide medical training in HK. Attitude towards Euthanasia (ATE), measured using a five-point Likert Scale, was used to assess medical students’ attitudes towards euthanasia. Results: overall, 228 valid responses were received in 2021. The mean score of ATE was 29 (SD10.9), in which 134 (58.8%) of respondents showed a negative attitude towards euthanasia. Negative association was found between Christian (*p*-value = 0.003) and Catholic (*p*-value = 0.032) and the ATE score. Meanwhile, positive association was found between male gender (*p*-value = 0.011) and witnessed withdrawing of nutritional support from patient(s) (*p*-value = 0.011) and the ATE score. Conclusions: It is necessary for the government and schools to place more emphasis on euthanasia in the school curriculum by integrating ethical discussions and clinical attachment.

## 1. Introduction

### 1.1. Background

Euthanasia (also known as “mercy killing”) means “to have a good death” in Greek [1]. Generally, euthanasia refers to ending a person’s life, especially terminally ill patients [2], with the aim to reduce the patient’s pain and suffering. There are two major forms of euthanasia: active euthanasia, where medical professionals take some measures to cause patients to die, such as providing lethal drugs and injections, and passive euthanasia, where medical professionals withhold life-sustaining treatments for patients, leading indirectly to their patient’s death [3]. The legislation of euthanasia first coalesced into an organized movement in England in 1935 [4]. Later, the Netherlands was the first country to legalize euthanasia (in 2001), followed by Belgium (in 2002) [4]. In recent decades, more and more jurisdictions have considered legalizing euthanasia. One of the possible reasons for this is the trend towards increasing individualization [5], and an aging population and medical advancement may also be important factors for advocating quality of life. In Australia, 50 attempts were made to legalize euthanasia before the voluntary euthanasia law was finally passed in 2017 [6]. Up to now, there are more than 20 jurisdictions that have legalized euthanasia [7]. However, many Western and Asian countries remain against legalizing euthanasia, including China, Denmark, and France [7]. The heated debate over euthanasia across the globe is worth our focus and consideration.

In Hong Kong, euthanasia has become a hot topic and public awareness on the issue has increased since the Tang Siu-pun (Ah-bun) case in the 2000s. Ah-bun mentioned that everyone has the right to control their lives and he demanded legislation to legalize euthanasia in 2004 after he was paralyzed in a gymnastics accident [8]. According to the Hospital Authority Guidelines on Life-Sustaining Treatment in the Terminally Ill, euthanasia is illegal and unethical [9]. However, it is legal to withdraw patients’ life-sustaining treatment under a specific condition, such as when all treatments are useless. Thus, euthanasia is worthy of our consideration as how and when people die is an important issue for patients and healthcare systems. With the rising aging population, more and more people are likely to suffer from chronic diseases such as stroke, dementia, and cancer. Under these conditions, patients are likely to have a poor quality of life due to prolonged suffering, especially for terminally ill patients. For example, they may have difficulty in swallowing and experience nausea [2]. Prolonged physical disability may cause them to feel depressed. Euthanasia, argue its proponents, can hope to reduce patients’ physical pain and persistent sufferings. In a study of 77 terminally ill patients in Hong Kong, 70% of them agreed with active euthanasia [10]. Apart from the importance for patients, euthanasia is also crucial in easing the heavy burden of the healthcare system. The cost of sustaining the life of terminally ill patients is huge and some doctors revealed that around 20–30% of expenses could be allocated to other healthcare provision if euthanasia is legalized [11]. 

There are many concerns associated with the decision to legalize euthanasia. Firstly, social culture and values are important to consider [12]. Many papers highlight that people in African countries are less supportive of euthanasia than Europeans [13]. One of the possible reasons is that Africans prefer controlling their own lives instead of “giving up”, and their beliefs and values of life affect their acceptance of euthanasia. More importantly, religion is another key factor regarding attitudes towards euthanasia. Countries with strong religious views based on Buddhism, Christianity, Islam, etc., most often strongly disapprove of euthanasia as they believe lives are god-sent and only God has the authority to decide when people are to die [14]. Some psychological factors (such as fear of dying) and education level (people with higher education tend to support euthanasia) possibly influence acceptance of euthanasia [15,16]. In addition to patients and the general population, assessing views from healthcare workers and students (healthcare professional-to-be) is equally important in understanding attitudes and the scope of management options for patient in the future.

As mentioned, euthanasia is a controversial topic across the globe. Research papers exist in other countries—such as in Canada [17], Sweden [18], South Africa [19], Mexico [20], Sri Lanka [21], etc.—on attitudes towards euthanasia among patients, physicians, doctors, nurses, and medical and nursing students. Some papers have studied the factors (such as religion, education level, psychological factors, etc.) affecting the acceptance of euthanasia. However, limited research papers have focused on the attitudes towards euthanasia among medical students with some associated factors, such as past clinical exposure and life experience. More importantly, among 20 research papers about euthanasia conducted in Hong Kong, there were no similar papers undertaken on the attitudes towards euthanasia among medical students. Instead, research only covered attitudes towards euthanasia among physicians [22] and terminally ill patients [10].

Medical students are future medical professionals, who will face many ethical dilemmas regarding requests for euthanasia by patients. Euthanasia may not be legal in Hong Kong, but how to respond to patients’ requests is still important. Hence, ascertaining their attitudes and factors affecting their choices, remains crucial for doctors when they perform or consider euthanasia for patients in the future. Considering different cultures, values, school curricula, and religions, this study was concerned with narrowing the knowledge gap and providing important input for the curriculum design. In doing so, it can provide useful insight into the readiness (in terms of psychological factors and knowledge) of medical students to consider and perform euthanasia when they become medical professionals in the future. With this in mind, exposure to euthanasia in the school curriculum and clinical setting can help medical students better inform government when they consider legalizing euthanasia. Thirdly, related training such as on moral education could prove useful to provide to medical students. Hence, medical students will then be well-prepared when they make decisions with their patients under different ethical parameters.

### 1.2. Aim and Objectives

This study aims to examine the attitudes of medical students towards euthanasia in Hong Kong and identify possible factors (religion, year of study, clinical exposure, gender, etc.) associated with their attitudes towards euthanasia.

## 2. Materials and Methods

### 2.1. Study Design

A cross-sectional online anonymous survey using a structured questionnaire format was conducted among medical students studying in two universities in Hong Kong. The study was approved by the committee of CUHK Survey and Behavioral Research Ethics before distributing the questionnaire. Moreover, participants were informed of the topic and objective on the first page of the questionnaire. Participants were free to end their involvement with the study at any time, and all questionnaires were anonymous and the collected data were stored confidentially.

### 2.2. Study Population

In Hong Kong, there are only two institutes—the University of Hong Kong (HKU) [23], and the Chinese University of Hong Kong (CUHK) [24]—that offer undergraduate medical programs. The CUHK offers the Medicine (MBChB) Program and the HKU offers a Bachelors of Medicine and Bachelors of Surgery (MBBS) Program. There were approximately 2780 medical students studying Year 1 to Year 6 in the CUHK and HKU in the 2019–2020 academic year [24]. We applied several inclusion and exclusion criteria for our research. Full-time undergraduate medical students from the CUHK (MBChB Program) and HKU (MBBS Program) were included in this study. In addition, all age, sex, and ethnic and racial groups were included, as these factors yielded a possible influence over their attitudes to euthanasia. Overseas exchange medical students were excluded as the education curriculum (about euthanasia) in Hong Kong was one of the study’s focuses. Furthermore, students who failed to complete the questionnaire and click the consent box were excluded from this study.

It is important to note that the population size was 2780 [25]. Assuming a 5% type I error and 52% proportion of students supporting euthanasia in a similar study [26], a sample size of 183 medical students is required to achieve 7% estimation precision (by accounting for the feasibility of data collection). By taking 20% missing data into account, the final sample size required for this study was rounded up to 229.

### 2.3. Data Collection

Participants were informed of the study topic on the first page of the online questionnaire. All personal information provided was anonymous and they could end the questionnaire at any time they felt uncomfortable. Participants could begin the questionnaire after they had clicked the consent box on page 1 of the online questionnaire.

The online self-administrated questionnaire was anonymous and conducted in English only, with definitions of some difficult terms such as active and passive euthanasia. The questionnaire was informed by a literature review and consisted of five parts with a total of thirty-six questions (open-ended questions were not counted): (1) Knowledge of euthanasia [27]; (2) Attitude towards Euthanasia (ATE) scale by Wasserman, Clair, and Ritchey, 2005 [26]; (3) Students’ exposure [26]; (4) Student’s family background [26]; and (5) Demographics. Participants were invited to finish the questionnaire within 10–15 min. A total of 15 eligible medical students from the CUHK and HKU were recruited to conduct the pilot test, which aimed at ensuring participants (regardless of their backgrounds) understood the questions provided. Amendments were thus made according to the participants’ views. For a better understanding of this study, a basic study overview was included on the first page of the online questionnaire. In addition, two reverse coding questions were included in the Attitude towards Euthanasia (ATE) scale to minimize bias. Lastly, all incomplete questionnaires were excluded from this study to ensure the quality and validity of data.

### 2.4. Questionnaire

In part one, the knowledge section included 4 questions aiming to test participants’ basic knowledge of euthanasia. The questions focused on whether students had heard of euthanasia and the different types of euthanasia. In addition, two True–False questions related to the current situation of euthanasia in Hong Kong were asked to reveal whether students would choose the right answer. In part two, 10 questions from the “Attitude towards Euthanasia (ATE) Scale” were used to examine students’ attitudes towards euthanasia [28]. Evidence showed that the ATE scale was both reliable and valid, with Cronbach’s alpha = 0.871 (Internal consistency) [26]. Many similar studies regarding attitudes towards euthanasia among medical students use the ATE scale as a reference [26]. The 10 questions were scored on a 5-point Likert scale (5 = strongly agree, 4 = agree, 3 = undecided, 2 = disagree, and 1 = strongly disagree). A higher score (4 or 5 marks) indicated a positive attitude towards euthanasia, while a lower score (1 or 2 marks) indicated a negative attitude. Reverse coding was used in Questions 6 and 9 of this scale. The marks from the 10 questions were summed up between 10 and 50 marks. The higher the total score, the more positive attitude held towards euthanasia, and vice-versa. In this study, 30 marks was used as the cut-off score [28]. Higher than or equal to 30 marks indicated a positive attitude towards euthanasia among medical students, while lower than 30 marks indicated a negative attitude.

Part three, on students’ exposures, included 12 questions (open-ended questions were not counted). Questions were related to students’ experience of witnessing the removal of life support treatment (both nutritional and mechanical) and some specific patients’ cases. In part four, family background included 4 questions related to some basic family information, such as parents’ occupation and education level. In the last part, demographics included 6 questions on ethnicity, gender, age, religion, education institute, and year of studies, etc.

All participants were recruited through convenience sampling via social media such as Facebook, Instagram, and WhatsApp. A Google form link to the questionnaire was included in the recruitment message. All Google form links were sent to the participants.

### 2.5. Data Processing and Analysis

Descriptive statistics were used to describe the questionnaire results. Statistics such as central tendency (mean), dispersion (standard deviations), and frequencies (with percentage) were shown. The descriptive statistics were applied on demographics (such as the year of studies, gender, and religion) and possible factors (such as students’ exposures and knowledge) associated with euthanasia.

Regarding the primary outcome (attitude towards euthanasia among medical students), the proportion of positive and negative attitudes towards euthanasia among medical students was analyzed. As mentioned above, the 10 questions from the ATE scale were summed up to between 10 and 50 marks. Students with mean ATE scores higher than or equal to 30 marks indicated positive attitudes towards euthanasia. Meanwhile, students with a mean ATE score lower than 30 marks indicated a negative and more oppositional attitude towards euthanasia.

Regarding the secondary outcome (factors associated with the attitude towards euthanasia), univariate and multivariate analyses were performed. For the univariate analysis, the *t*-test (for two groups) or ANOVA (for more than two groups) was used to analyze the associations between each factor (including demographics, knowledge, student exposures) and the ATE score. A *p*-value smaller than 0.05 indicated a significant association between the specific factor and the ATE score. Those factors with a *p*-value smaller than 0.2 were kept and then underwent multivariate analysis. For multivariate analysis, multiple linear regression was used to identify which factors were associated with the ATE score. *p*-values smaller than 0.05 were considered statistically significant.

In this study, Microsoft Excel was used to summarize the descriptive data and to clean data, while R (Version 4.0.3) for Windows was used for data analysis. The confidence interval used for this study was 95% and a *p*-value smaller than 0.05 indicated a significant association.

## 3. Results

### 3.1. Demographics

A total of 236 participants completed the questionnaire between June and September 2021, which fulfilled the minimum sample size calculated above. However, 8 questionnaires containing missing data and thus 228 valid questionnaires were used for data analysis. The majority of participants were female (57%), from the CUHK (82%), studying pre-clinical year (Year 1–3) (73.2%), and Chinese (98.3%). Most of the participants were Atheist, 113 (49.6%), followed by Christian 66 (28.9%), and Catholic 18 (7.9%). A minority of participants, 31 (13.6%), practiced other religions (including Buddhist, Hindu, Muslim, etc.). Age was quite even in both age groups (Table 1).

The mean ATE score was significantly higher in male (*p* = 0.02), HKU (*p* = 0.015), and atheist (*p* = 0.028) than their counterparts. No significance was observed for age group, study year, or ethnicity.

### 3.2. Attitude towards Euthanasia

Among the 228 participants, the mean score of Attitude towards Euthanasia (ATE) was 28.9 (SD10.9) (Table 1). A total of 134 respondents (58.8%) gave lower than 30 marks, which indicated negative attitudes and opposition towards euthanasia, whereas 94 (41.2%) of the total participants gave higher than or equal to 30 marks, indicating a positive attitude towards euthanasia.

### 3.3. Knowledge, Exposure, and Readiness Related to Euthanasia

Around 46.1% of participants possessed good knowledge of euthanasia, demonstrated by answering all four items correctly (Table 2). However, there was no significant association between knowledge and attitude towards euthanasia (*p* = 0.134). Clinical exposure in terms of “witnessed withdrawing of nutritional support from patients” was found significantly correlated with a positive attitude towards euthanasia (*p* = 0.018). However, no significance was found in lecture-based exposure to euthanasia and readiness to assist decision making on euthanasia (*p* > 0.05).

### 3.4. Factors Associated with Attitudes towards Euthanasia

As mentioned in the methodology, factors with *p*-value smaller than 0.2 in the univariate analysis were kept and underwent multivariate analysis (Table 3). Male gender (*p*-value = 0.011), Christianity (*p*-value = 0.001), and having witnessed withdrawing of nutritional support from patient(s) (*p*-value = 0.011) were significant. Males had a mean ATE score 2.7 marks higher than female (adjusted coefficient 2.7; 95% CI 0.5 to 4.8; *p*-value = 0.017). Christianity had a mean ATE score 3.8 marks lower than Atheist (adjusted coefficient −3.7; 95% CI −6.2 to −1.2; *p*-value = 0.003). Students who had other religions had a mean ATE score 1.4 marks lower than Atheist; however, this was not statistically significant. Students who witnessed the withdrawal of nutritional support from patient(s) had a mean ATE score 4.6 marks higher than those who did not (adjusted coefficient 4.6; 95% CI 0.8 to 8.4; *p*-value = 0.017).

## 4. Discussion

From the results, about 58% of medical students did not support euthanasia in this study. This is a similar observation to other jurisdictions with negative attitudes towards euthanasia, shown at 52% in Poland (Positive attitude: 12%; Not sure: 37%) [29], 52% in Switzerland (Positive attitude: 34%; Not sure: 13%) [18], 57% in Germany (Positive attitude: 19.2%; Not sure: 21.8%) [30], 61% of medical students in Malaysia (Positive attitude: 39%) [26], and 76% in Norway (Positive attitude: 24%) [31]. Although a few countries such as India (Negative attitude: 40%; Positive attitude: 60%) [32] showed opposite results to this study, most of the medical students in other countries showed as negative attitudes towards euthanasia as shown by medical students in Hong Kong. By viewing different research papers conducted around the world, it is clear that many medical students showed a negative attitude towards euthanasia. This raises the question of whether legalizing euthanasia is a good move. Many factors (such as religion and clinical exposures) were related to the attitudes towards euthanasia among medical students.

In this study, several factors were significantly associated with the mean ATE score. Firstly, Christian (*p*-value = 0.003) and Catholic (*p*-value = 0.032) showed negative association with the mean ATE score. Christians, including Christians and Catholics, exhibited relatively negative attitudes towards euthanasia compared to non-Christians. Many previous studies showed the same result, such as in Karachi [27] and Poland [29]. The negative association between religion and attitude towards euthanasia could be explained by the cultural factors and ethical views promoted by different religions [27]. The literature review highlighted that people with religious beliefs preached that life is god-sent, that is, life considered as a gift [27]. Only God has our life in his hands. Hence, some religions such as Catholicism and Christianity treat euthanasia as murder. More specifically, a study in Poland [29] revealed that 85% of the medical students were Catholics and most of them exhibited negative attitudes towards euthanasia. According to the research, their decisions were affected by the Catholic Church and statements made by Jesus Christ [29]. Belgium, as an example of a non-Christian country, legalized euthanasia. They are more open to discussing death and respecting choices about death, and they believe that they have the rights to control their own life and death, not only for adult, but also children [33]. These findings supported the negative attitudes towards euthanasia among Christians and Catholics compared to non-Christians and non-Catholics. The religious background of medical students in Hong Kong can be a possible reason mediating their acceptance or rejection of euthanasia. Thus, religion is one of the crucial factors to be considered when deciding on whether to legalize euthanasia in Hong Kong.

Second, male gender (*p*-value = 0.011) showed a positive association with the mean ATE score, thus demonstrating a relatively positive attitude towards euthanasia compared to females. Several studies have revealed the same result [29,34]. There are few papers that explain this gender difference among medical students, however, a study explained the gender difference among physicians and found that female physicians were more vulnerable when facing a dying or incurably ill patient than male physicians [35]. Females showed a more emotional response and found it harder to make decisions when considering ending a patient’s life. This finding could help to explain why female medical students showed relatively less support for euthanasia than males. It is worth considering whether some emotion management courses could be provided to female medical students in the school curriculum, which may affect their views towards euthanasia.

Besides that, witnessed withdrawing of nutritional support from patient(s) (*p*-value = 0.011) was also positively associated with the mean ATE score. Thus, medical students that witnessed withdrawing of nutritional support from patient(s) showed relatively positive attitudes towards euthanasia compared to those who did not. A study conducted in Karachi showed that medical students with more clinical exposures (such as having witnessed withdrawing of medical support) also demonstrated the same trend [27]. Since some incurable diseases were associated with patients’ sufferings on a physical, emotional, and psychological level [27], euthanasia could be a relief to some patients. Hence, when medical students had witnessed withdrawing of nutritional support from patients, they were more likely to support euthanasia than those who had not. These finding show that clinical exposure is a crucial factor influencing medical students’ acceptance of euthanasia or not.

Although the university curriculum teaching on euthanasia proved insignificant (*p*-value = 0.064) in this study, it is worthy of further discussion. In the open-ended questions, many medical students reflected that the school curriculum had little coverage of euthanasia, with little or no opportunity to discuss and reflect on this topic. Many medical students hope to have more lectures covering euthanasia, so they can make a decision with relevant knowledge and also have more understanding from the perspective of patients and their family caregivers.

### Strengths and Limitations, and Further Investigation

This research constituted the first study to provide insights on attitudes towards euthanasia among medical students in Hong Kong. Indeed, there are difficulties in conducting similar studies in Hong Kong due to the cultural taboo for locals to discuss death. Hong Kong people tend to have strong traditional cultural beliefs about death because they believe taking about death can bring bad luck [33,36]. Some of them even have anxiety when they come across death preparation, which involves psychological preparedness, balance between preference and feasibility, family responsibility, and financial burden [37,38]. It is challenging to design the depth and breadth of the after-death issues in the medical curriculum, but it is important to have further study or research work on the topic of life and death. Due to the consideration of length of questionnaire, not all of the factors related to euthanasia were included in this study, however, most of the significant factors, such as religion, knowledge, student exposures, etc., suggested by similar studies, were included.

However, this study does have some limitations. Firstly, it used the Attitude towards Euthanasia (ATE) scale and had only 10 questions for this scale, which could not capture all possible ways that the participants might support or not support euthanasia [26]. For example, family factors were excluded from the scale. Some case-study scenarios with different possible variations (such as family and patients’ health conditions) can be provided in future studies [39]. Complementary scales can be used to assess attitudes towards euthanasia. Furthermore, the views of ordinary citizens on euthanasia were not included in the survey, and are important to investigate in case there is any weight-bias between a doctor’s own view and patient’s preference. Moreover, since the total sample is 228, it possibly limited statistical power to categorize the ATE outcome into three groups (positive, uncertain, did not support) and test their association with factors from demographics, knowledge, curriculum, clinical exposure, and readiness.

Additionally, we cannot find causal relationships in this cross-sectional study. Further studies including longitudinal studies could be used to discern this. Indeed, there are some pre-existing longitudinal papers related to euthanasia, such as “A longitudinal study of attitudes toward physician-assisted suicide and euthanasia among patients with non-curable malignancy” [40]. Meanwhile, similar qualitative research can be performed among medical students in the future to study their change in attitudes. Hence, readers can have a better understanding of the psychological factors (such as empathy level) related to the attitude towards euthanasia from the stage of professional-to-be to professional-in-practice.

Lastly, this study only showed whether medical students had positive or negative attitudes towards euthanasia; however, the degree of acceptance towards euthanasia could not be fully reflected. Meanwhile, different types of euthanasia (voluntary active, voluntary passive, involuntary active, involuntary passive) were considered as a whole in this study. However, participants might have different views towards different types of euthanasia. For further studies, we suggest disaggregating different types of euthanasia and studying the degree of acceptance towards euthanasia.

## 5. Conclusions

In conclusion, more than half of Hong Kong medical students did not support euthanasia. Besides gender and religious belief, clinical exposure was found significantly associated with attitudes towards euthanasia. The level of awareness of euthanasia has increased in recent years. Medical students are future medical professionals who will face many ethical dilemmas, particularly in palliative care, hospice care, and issues of euthanasia. It is crucial to equip them with knowledge and clinical experiences to consider euthanasia from an informed position. Therefore, we suggest strengthening of the curriculum and training in order for medical students to have an informed ethical position on this topic. We suggest further studies about euthanasia and quality of death, medical students can then better understand and empathize in future co-decision making with the patients and their families on the medical, legal, and ethical aspects of euthanasia.

## Figures and Tables

**Table 1:** Demographics of undergraduate medical students (*n* = 228).

Demographics Characteristics	*n* (%)	Attitude towards Euthanasia Mean Score (SD)	*p*-Value ^&^
Age			0.884
17–19	111 (48.7)	28.5 (8.6)	
20–25	117 (51.3)	28.3 (8.1)	
Gender			0.002 *
Male	98 (43.0)	30.4 (9.1)	
Female	130 (57.0)	26.9 (7.4)	
Institute			0.015 *
CUHK ^^^	187 (82.0)	27.8 (7.8)	
HKU ^^^	41 (18.0)	31.3 (10.1)	
Academic Year			0.202
Basic Year (Year 1–3)	167 (73.2)	28.0 (8.3)	
Advanced Year (Year 4–6)	61 (26.8)	29.6 (8.4)	
Ethnicity			0.496
Chinese	224 (98.3)	28.4 (8.4)	
Non-Chinese	4 (1.8)	4 (5.5)	
Religion			0.028 *
Atheist	113 (49.6)	30.0 (8.9)	
Christianity	84 (36.8)	26.4 (7.0)	
Other	31 (13.6)	28.1 (8.6)	
All respondents	228 (100.0)	28.9 (10.9)	N/A

^^^ CUHK—The Chinese University of Hong Kong; HKU—The University of Hong Kong. ^&^ Either *t*-test or ANOVA was performed. * *p*-value was significant at <0.05.

**Table 2:** Univariate Analysis of KAP (*n* = 228).

Factors Affected Attitude	*n* (%)	Attitude towards Euthanasia Mean Score (SD)	*p*-Value
Knowledge of Euthanasia			0.134
Good (Passed all four knowledge questions)	105 (46.1)	27.5 (8.0)	
Poor	123 (53.9)	29.2 (8.6)	
Academic Exposure on Euthanasia			0.053
Sufficient university curriculum on euthanasia			
Yes	118 (51.8)	27.4 (7.6)	
No	110 (48.2)	29.5 (9.0)	
Personal Exposure of Euthanasia			
(1) Family member(s) with life support treatment			0.754
Yes	86 (37.7)	28.2 (8.8)	
No	142 (62.3)	28.6 (8.1)	
(2) Accompanied a dying person to the end			0.653
Yes	66 (28.9)	28.0 (9.0)	
No	162 (71.7)	28.6 (8.1)	
(3) Witnessed withdrawing of mechanical support from patient(s)			0.576
Yes	28 (12.3)	29.2 (9.9)	
No	200 (87.7)	28.3 (8.1)	
(4) Witnessed withdrawing of nutritional support from patient(s)			0.018 *
Yes	21 (9.2)	32.5 (10.0)	
No	207 (90.8)	28.0 (8.1)	
Readiness to assist decision making on Euthanasia			
(1) In terms of knowledge			0.246
Yes	89 (39.0)	27.6 (7.2)	
No	139.0 (61.0)	28.9 (9.0)	
(2) In terms of psychological conditions			0.499
Yes	118.0 (51.8)	28.1 (7.5)	
No	110.0 (48.2)	28.8 (9.2)	

* *p*-value < 0.05 (significant).

**Table 3:** Multiple Linear Regression of different factors associated with the ATE score (demographics adjusted) (*n* = 228).

Covariate	Coefficient (95% CI)	*p*-Value
Constant	31.2 (28.1, 34.4)	<0.001
Knowledge of Euthanasia		0.228
Poor	Ref	
Good	−1.3 (−3.4, 0.8)	
Personal Exposure of Euthanasia		0.015 *
Not witnessed withdrawing of nutritional support from patient(s)	Ref	
Witnessed withdrawing of nutritional support from patient(s)	4.6 (0.9, 8.4)	
Academic Exposure of Euthanasia		0.149
Insufficient university curriculum on euthanasia	Ref	
Sufficient university curriculum on euthanasia	−1.6 (−3.8, 0.6)	
Demographics		
*Gender*		0.015 *
Female	Ref	
Male	2.7 (0.5, 4.8)	
*Institute*		0.254
HKU ^^^	Ref	
CUHK ^^^	−1.7 (−4.5, 1.2)	
*Religion*		
Atheist	Ref	
Christianity	−3.8 (−6.1, −1.5)	0.001 *
Other	−1.4 (−4.6, 1.8)	0.403

^^^ HKU—The University of Hong Kong; CUHK—The Chinese University of Hong Kong. * *p*-value < 0.05 (significant). Remarks: all are dummy variables.

## Data Availability

The datasets are available from the corresponding author on reasonable request. The data are not publicly available due to their containing information that could compromise the privacy of research participants.
